# Increase in teledermatology consultations regarding suspected tinea capitis – an observation of misidentified nevi flammei

**DOI:** 10.1111/ddg.15896

**Published:** 2025-09-19

**Authors:** Julian Kött, Johannes Dupont, Katharina Langen, Christian Drerup

**Affiliations:** ^1^ Department of Dermatology and Venereology University Medical Center Hamburg‐Eppendorf Hamburg Germany; ^2^ doctorderma – Online Dermatology Hamburg Germany; ^3^ Department of Dermatology Venereology and Allergology University Hospital Schleswig‐Holstein – Campus Kiel Kiel Germany

Dear Editors,

We have observed consultations submitted to our teledermatology service *doctorderma* by patients expressing concern about the possible development of tinea capitis on the scalp. In Q3/24, however, the condition was increasingly not a fungal infection but rather a benign nevus flammeus (port‐wine stain), typically located on the back of the neck.

Tinea capitis has attracted increasing attention from dermatologists worldwide and in Germany since 2023.^1^, ^2^, ^3^, ^4^ To date, *Microsporum canis*, *Trichophyton mentagrophytes*, and *Trichophyton benhamiae* were the three most frequent pathogens reported in tinea capitis in Germany, but *Trichophyton tonsurans* continues to spread. A single‐center observation at the Technical University of Munich reported an increase in Trichophyton tonsurans in tinea capitis cases, from 33.3% (2019–2021) to 68.8% in 2022.^2^ In our online clinic, tinea capitis accounted for between 0.1% (n = 1) of all diagnoses in Q1/2023 and 0.58% (n = 11) in Q2/2023 (Table 1).

**TABLE 1:** Percentage of tinea capitis and nevi flammei of the head and neck.

	Q 1/23	Q 2/23	Q 3/23	Q 4/23	Q 1/24	Q 2/24	Q 3/24
n (inquiries related to tinea capitis)	0.10% (1)	0.58% (11)	0.64% (15)	0.59% (17)	0.55% (17)	0.50% (25)	0.87% (50)
n (tinea capitis)/ n (total)	0.10% (1)	0.58% (11)	0.60% (14)	0.52% (15)	0.52% (16)	0.46% (23)	0.66% (38)
n (nevi flammei)/ n (total)	0 (0)	0 (0)	0.04% (1)	0.07% (2)	0.03% (1)	0.04% (2)	0.21% (12)

*Abbr*.: Q, quarter

The observation that patients contact us directly with the question of a “fungal infection after a visit to the barbershop” correlates strongly with (social) media coverage of the issue. Major daily newspapers reported on the problem for the first time in July 2024 (spiegel.de, “Spreading of fungal skin infections due to trend hairstyles,” July 6, 2024; bild.de, “Dangerous fungal skin infection! Doctors warn against trendy hairstyles,” July 15, 2024). In social media, the topic had already emerged in April 2024, in particular in TikTok and Instagram videos that were viewed millions of times. These reports, although well intentioned, appear to have contributed to an increase in dermatological consultations driven by fear of tinea capitis.

In the vast majority of cases, patients reported noticing a reddish or pinkish patch on the back of their head that they had never seen before. Most often, this coincided with a recent haircut, particularly styles that expose the nape of the neck, such as a close shave or a so‐called “fade.” While the nevus flammeus may have been present since birth, it often goes unnoticed until the hair covering the area is significantly shortened. Discovery of the patch alarms many patients, who fear that they have contracted tinea capitis.

The reality, however, is that while tinea capitis is a well‐known scalp condition in children and adults,^3^ it typically presents with more distinctive symptoms, including hair loss, scaly patches, and itching, none of which are typically associated with a nevus flammeus.^4^, ^5^

Since Q1/2023, we have reviewed 136 cases in which patients, following a haircut, submitted images of the nape of the neck, believing they had developed a fungal infection. This suspicion was explicitly stated in all submissions.

In 13.2% (18 of 136 suspected cases), our dermatologists could confirm by teledermatology that the lesions were congenital vascular birthmarks, known as port‐wine stains, which are completely benign and not a cause for concern. The proportion of these congenital cases increased from 0.04–0.07% (total n = 6) between Q3/2023 and Q2/2024 to 0.21% (total n = 12) in Q3/2024 (Table 1). This trend appears to be closely linked to the current media narrative surrounding barbershop‐associated fungal infections.

Patients seem to be increasingly vigilant about changes to their skin, particularly following haircuts. Overall, this awareness has a positive impact on dermatological health, especially regarding potentially infectious diseases. Even though the cases described here were misinterpreted, early medical education might have helped prevent the wider transmission of tinea capitis. The educational potential of both traditional and social media is striking. However, this surge in consultations regarding tinea capitis serves as a reminder that media‐driven health scares can sometimes lead to widespread confusion and the misidentification of benign conditions.

In conclusion, we encourage our colleagues in both dermatology and the media to collaborate in ensuring that public health messages are conveyed responsibly and that patients are empowered with accurate information. In particular, alongside the risk of tinea capitis, possible differential diagnoses such as nevi flammei should be highlighted in print media and on social networks.

## CONFLICT OF INTEREST STATEMENT

JK and KL recieved honoraria from doctorderma‐Online Hautarzt. JD is an employee of doctorderma‐Online Hautarzt. CD is founder and CEO of doctorderma‐Online Hautarzt.
